# TLDc Domain-Containing Genes in Autism Spectrum Disorder: New Players in the Oxidative Stress Response

**DOI:** 10.3390/ijms242115802

**Published:** 2023-10-31

**Authors:** Cinzia Zucchini, Carmela Serpe, Paola De Sanctis, Alessandro Ghezzo, Paola Visconti, Annio Posar, Federica Facchin, Marina Marini, Provvidenza Maria Abruzzo

**Affiliations:** 1 Department of Medical and Surgical Sciences, University of Bologna, Via Massarenti 9, 40138 Bologna, Italy; cinzia.zucchini@unibo.it (C.Z.); carmela.serpe@unibo.it (C.S.); paola.desanctis@unibo.it (P.D.S.); federica.facchin2@unibo.it (F.F.); provvidenza.abruzzo2@unibo.it (P.M.A.); 2 Grioni Center-Danelli Foundation, Largo Stefano ed Angela Danelli 1, 26900 Lodi, Italy; a.ghezzo@fondazionedanelli.org; 3 IRCCS Istituto delle Scienze Neurologiche di Bologna, UOSI Disturbi dello Spettro Autistico, Via Altura 3, 40139 Bologna, Italy; paola.visconti@ausl.bologna.it (P.V.); annio.posar@unibo.it (A.P.); 4 Department of Biomedical and Neuromotor Sciences, University of Bologna, Via Altura 3, 40139 Bologna, Italy

**Keywords:** TLDc domain-containing proteins, autism spectrum disorder, inflammation, oxidative stress, OXR1, TLDC1, NRF2, TUG1, lncRNAs, miRNAs

## Abstract

Oxidative stress (OS) plays a key role in autism spectrum disorder (ASD), a neurodevelopmental disorder characterized by deficits in social communication, restricted interests, and repetitive behaviors. Recent evidence suggests that the TLDc [Tre2/Bub2/Cdc16 (TBC), lysin motif (LysM), domain catalytic] domain is a highly conserved motif present in proteins that are important players in the OS response and in neuroprotection. Human proteins sharing the TLDc domain include OXR1, TLDC1, NCOA7, TBC1D24, and C20ORF118. This study was aimed at understanding whether TLDc domain-containing mRNAs together with specific microRNAs (200b-3p and 32-5p) and long noncoding RNAs (TUG1), known to target TLDc proteins, contributed to regulate the OS response in ASD. Data showed a significant increase in the levels of *OXR1* and *TLDC1* mRNAs in peripheral blood mononuclear cells (PBMCs) of ASD children compared to their neurotypically developing (NTD) counterparts, along with an increase in TUG1 mRNA expression levels, suggesting its possible role in the regulation of TLDc proteins. A positive correlation between the expression of some TLDc mRNAs and the Childhood Autism Rating Scale (CARS) global score as well as inflammatory gene expression was found. In conclusion, our data suggest a novel biological pathway in the OS response of ASD subjects that deserves further exploration.

## 1. Introduction

Autism spectrum disorder (ASD) is a complex and biologically based neurodevelopmental disorder characterized by a spectrum of symptoms ranging in severity from mild to severe involving impairment in social communication, repetitive behaviors, and restricted interests [1,2]. In addition to these core symptoms, ASD subjects experience a range of neuropsychiatric and medical comorbidities, including intellectual disability, attention-deficit/hyperactivity disorder (ADHD), anxiety, depression, epilepsy, gastrointestinal and immune dysfunctions, and metabolic disorders [3,4,5,6,7,8]. The prevalence of ASD worldwide has been increasing over the last two decades and has been recently reported to be 1:36 in 8-year-olds in the USA. In Italy, the estimated prevalence of ASD is 1:77 according to the Ministry of Health [9]. The male to female prevalence ratio is approximately 3.8:1 [2]. Although there are no reliable biomarkers, ASD is clinically diagnosed around the age of 3 years [10] using standardized diagnostic tools, such as the Autism Diagnostic Observation Schedule (ADOS), the Childhood Autism Rating Scale (CARS), or the Autism Diagnostic Interview-Revised (ADI-R) to assess children’s behavior and symptoms [11,12]. To date, pharmacological interventions aim to control the symptomatology associated with ASD comorbidities, but there are no therapeutic drugs targeting the core symptoms [13].

Despite its increasing prevalence, the pathophysiology of ASD remains unclear and incomplete. Strong genetic evidence suggests that ASD etiology involves the mutation, inactivation, or dysregulation of several hundreds of genes, with environmental and epigenetic factors playing a role in their impairment or dysfunction [14,15]. Additionally, oxidative stress (OS) has been proposed to play a crucial role in ASD [16,17,18]. OS results from an imbalance between reactive oxygen/nitrogen species (ROS/RNS) production and the antioxidant defense system, which is mainly based on enzymes and molecules able to counteract and mitigate the deleterious effects of ROS/RNS [19]. Such imbalance can cause damage to DNA, proteins, and lipids and ultimately may lead to cell death [19]. Several authors have reported an increase in ROS levels and a reduction in antioxidant capacity in ASD patients not only in the brain but also at the systemic level, highlighting the involvement of OS in ASD [15,17,18,20,21,22,23,24]. An increase in OS is strongly connected to alterations in immune system, inflammation, and mitochondrial dysfunctions that have already been described in ASD patients, which, in a kind of vicious circle, reinforce one another [15,18,20,25,26]. Moreover, OS appears to affect the expression of genes involved in ASD pathophysiology [27]. An increased burden of de novo or rare CNVs (copy number variations) has been reported in ASD patients; most CNVs are located in specific loci, including those associated with “genes of the cellular response to reactive oxygen species’’ (Gene Ontology ID: 0000302), suggesting a particular importance in dysregulation of the antioxidant defense system in this disorder [28,29]. The manipulation of OS during development allows the demonstration of its role in ASD pathogenesis in animal models [30]; in humans, a role of OS can be inferred only by observing the mitigation of ASD-typical behaviors by treatment with antioxidants or anti-inflammatory compounds [30]. Moreover, the level of urinary 8-isoprostane, an oxidative damage marker, was found to directly correlate with cognitive impairment in ASD children [24]. A better characterization of OS molecular pathways and the identification of novel antioxidant proteins could be useful for designing new therapeutic strategies to counteract damaging ROS in ASD patients.

Recently, a new family of proteins associated with oxidation-related functions was described [31]. These proteins share a highly conserved motif, named TLDc [Tre2/Bub2/Cdc16 (TBC), lysin motif (LysM), domain catalytic], hence their designation as “TLDc domain-containing proteins”. In humans, the TLDc domain-containing gene family is composed of five genes: oxidation resistance 1 (*OXR1* or *TLDC3*), TBC/LysM-associated domain containing 1 (*TLDC1* or *KIAA1609*), nuclear receptor coactivator 7 (*NCOA7* or *TLDC4*), TBC1 domain family member 24 (*TBC1D24* or *TLDC6*), and TBC/LysM-associated domain containing 2 (*C20ORF118* or *TLDC2*) [31]. For each gene, several isoforms have been described, including some short isoforms that only consist of the C-terminal TLDc domain [31,32]. The most established biological property common to all members of the TLDc protein family is a protective function against OS, conferred by the TLDc domain through molecular mechanisms that remain to be elucidated [32]. This function allows TLDc proteins to mitigate the effects of ROS in neurological diseases characterized by the involvement of OS [31,32,33,34]. In fact, it was demonstrated that OXR1 plays a protective role in oxygen-induced retinopathy, diabetic retinopathy, Parkinson’s disease, ischemia-induced neuronal damage, and amyotrophic lateral sclerosis (ALS) [35,36,37,38,39,40,41]. In addition, mutations in the TLDc proteins have been reported to be associated with several neurological diseases [32]. For instance, loss-of-function mutations in TBC1D24 have been described in a range of human diseases, most of which are associated with epileptic seizures [42]. Moreover, biallelic loss-of-function mutation in OXR1 is associated with autosomal-recessive neurological disease with cerebellar atrophy and lysosomal dysfunction characterized by developmental delay, intellectual disability, hypotonia, epilepsy, and cerebellar anomalies. Interestingly, these severe loss-of-function and null mutations can be rescued with a single short OXR1 cDNA containing only the TLDc domain [43]. As for NCOA7, a biallelic loss-of-function variant was described in one ASD male subject [44], while mice lacking this gene exhibit alterations in neuronal development and social behavior. Interestingly, NCOA7 mutations mainly affect lysosomal functions in neurons [45]. 

This study aimed to explore the potential involvement of TLDc family genes in idiopathic ASD and to assess the contribution of noncoding RNAs in their regulation. In particular, miR-200b-3p, miR-32-5p, and long noncoding RNA taurine up-regulated 1 (TUG1), which are known to target the TLDc domain-containing proteins, were evaluated. Furthermore, correlations between ASD severity and OS- and inflammation-related gene expression were studied. Our study defines the expression profiles of these genes in the context of OS and immune activation associated with ASD, paving the way for the discovery of new potential biomarkers for diagnosis and therapy.

## 2. Results

### 2.1. Expression of Genes Coding for TLDc Domain-Containing Proteins

The expression of genes coding for the TLDc domain-containing proteins *OXR1*, *TLDC1*, *NCOA7*, *TBC1D24*, and *C20ORF118* was evaluated in peripheral blood mononuclear cells (PBMCs) isolated from children with ASD and from their neurotypically developing (NTD) counterparts using quantitative real-time PCR (qPCR). 

**Figure 1:**
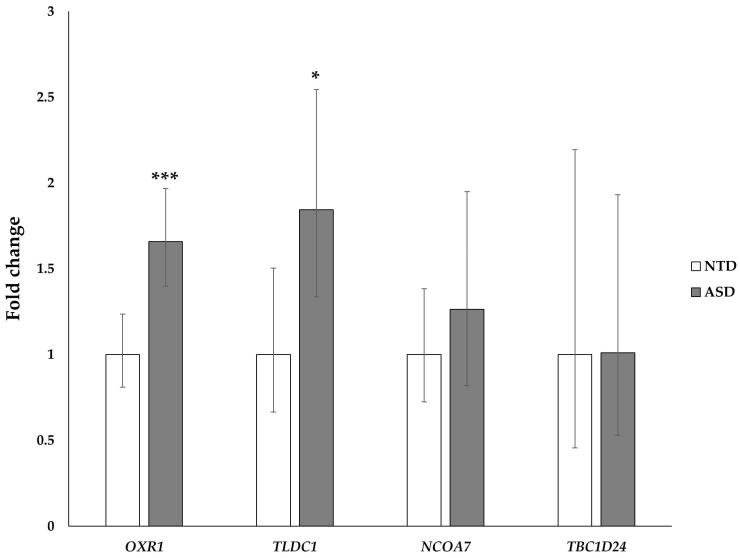
Expression of genes coding for TLDc domain-containing proteins in peripheral blood mononuclear cells (PBMCs) isolated from autism spectrum disorder (ASD) and neurotypically developing (NTD) children. For each gene, the mean expression value of the ASD group is compared to the mean expression value of the NTD group, set at 1. *OXR1*, oxidation resistance 1; *TLDC1*, TBC/LysM-associated domain containing 1; *NCOA7*, nuclear receptor coactivator 7; *TBC1D24*, TBC1 domain family member 24. The data are reported as the fold change ± the 95% confidence interval. (***) *p* ≤ 0.001; (*) *p* ≤ 0.05.

Genes coding for TLDc domain-containing proteins were expressed in both NTD and ASD PBMCs, with the only exception of *C20ORF118*, which was below the detection limit in all the examined samples. As shown in Figure 1, a trend of an increase in the mRNA abundance of *OXR1*, *TLDC1*, and *NCOA7* was observed in the ASD group compared to the NTD group; this increase reached statistical significance for the *OXR1* and *TLDC1* genes, even when the Benjamini and Hochberg false discovery rate (FDR) test was applied (*OXR1* pFDR = 0.0031; *TLDC1* pFDR = 0.042). In particular, the increase in the *OXR1* expression level was observed in 93.75% of ASD children (15 children out of 16), while the increase in the *TLDC1* expression level was observed in 87.5% of ASD children (14 children out of 16).

### 2.2. miR-200b-3p, miR-32-5p, and lncRNA TUG1 Expression

**Figure 2:**
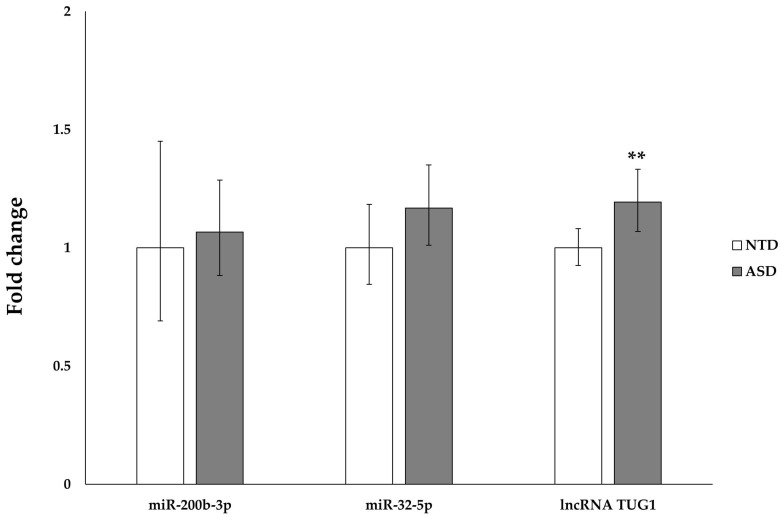
miR-200b-3p, miR-32-5p, and lncRNA TUG1 expression levels in peripheral blood mononuclear cells (PBMCs) isolated from autism spectrum disorder (ASD) and neurotypically developing (NTD) children. For each gene, the mean expression value of the ASD group is compared to the mean expression value of the NTD group, which was set at 1. TUG1, taurine upregulated 1. Data are reported as the fold change ± the 95% confidence interval. (**) *p* ≤ 0.01.

To help elucidate the molecular mechanisms underlying the differential expression of *OXR1* and *TLDC1* in ASD vs. NTD children, we focused on specific noncoding RNAs. Through a systematic search of published data, two microRNAs, miR-200b-3p and miR-32-5p, were selected since they target OXR1 and TLDC1, respectively [36,46] and are both expressed in PBMCs [47,48]. No difference in the expression levels of either miRNA was detected between ASD and NTD subjects (Figure 2), suggesting that these miRNAs are not involved in the upregulation of *OXR1* and *TLDC1* observed in ASD PBMCs (Figure 1). In addition to the abovementioned miRNAs, we analysed the expression level of TUG1 lncRNA, previously detected in the peripheral blood of autistic patients and reported as a positive regulator of *TLDC1* [49,50]. In ASD PBMCs compared to NTD PBMCs, we found a slight, yet significant, increase in the TUG1 lncRNA level (pFDR: 0.033) (Figure 2).

### 2.3. TLDc Family Gene Expression: Correlation between Clinical Features and Inflammation/Oxidation-Related Genes

The parametric (Pearson) test was used to correlate clinical features (CARS global scores, Table S1) or inflammation/oxidation-related gene expression data to TLDc family gene profiles. In the ASD group, the levels of *OXR1*, *TLDC1*, and *TBC1D24* mRNAs positively correlated with the CARS global scores (Table 1), indicating connections between the expression levels of these genes and the severity of the condition.

To highlight the relationships between the TLDc family proteins and inflammation/oxidative stress pathways, we utilized our previously published data on the same group of patients [20,51]. In particular, we focused on the mRNA expression levels of genes chosen for their relevant role in inflammatory processes or in redox state balance. Overall, a positive correlation was observed between the expression levels of TLDc domain-containing mRNAs and those of mRNAs encoding inflammation-related proteins, i.e., interleukin 1 beta (*IL1B*), tumor necrosis factor-alpha (*TNF-alpha*), cyclooxygenase 2 (*COX2*), and aryl hydrocarbon receptor (*AHR*) (Table 2). Over half of the correlations were statistically significant; after applying the FDR test, some correlations lost statistical significance (*p* > 0.05); however, authoritative scholars [52,53] have argued that setting a more liberal threshold (as high as 0.1 or even a bit higher) may be reasonable for pFDR. In summary, our results point to a possible functional connection between TLDc domain-containing proteins and proinflammatory pathways. Then, the expression levels of TLDc domain-containing mRNAs were evaluated in relation to the expression profile of cytoprotective and antioxidant genes, peroxiredoxin 2 (*PRDX2*), and nuclear factor erythroid 2-related factor 2 (*NRF2*). A positive correlation was found between *NRF2* and three members of the TLDc family genes, *TLDC1*, *NCOA7*, and *TBC1D24*. Conversely, no correlation was observed for *PRDX2*.

## 3. Discussion

Recent studies highlighted the neuroprotective and antioxidant role of a family of proteins characterized by the presence of the so-called TLDc [Tre2/Bub2/Cdc16 (TBC), lysin motif (LysM), domain catalytic] domain [32]. Several pieces of evidence demonstrate that the TLDc family proteins are essential for the development and proper functioning of the brain [31,32,54,55]. It has been reported that loss of function of OXR1, NCOA7, and TBC1D24 is associated with neurological diseases, including ASD, characterized by aberrant neurodevelopment [42,43,44,45]. A recent study focused on the role of the TLDc domain in patients with several neurological anomalies carrying a loss-of-function variant of the TLDc domain of OXR1; by using patient-derived brain organoids, authors modelled the function of OXR1 in early brain development, revealing that OXR1 contributes to the spatial-temporal regulation of histone arginine methylation in specific brain regions [56]. The protective role of TLDc family proteins in OS-dependent neurodegenerative diseases has also been described [31,32,57]. The overexpression of OXR1 delayed the occurrence of oxidative damage in the spinal cord and improved motor neuron survival, motor coordination, and life span in amyotrophic lateral sclerosis mice [38], suggesting that elevated OXR1 levels play a protective role. The overexpression of TLDC1 in mammalian neurons was shown to be protective against OS [31]. These biological features prompted us to analyze for the first time the mRNA expression levels of the TLDc family proteins in PBMCs isolated from ASD children. A trend of an increase in the levels of *OXR1*, *TLDC1*, and *NCOA7* mRNAs was observed in ASD children compared to NTD children, although the increase reached statistical significance only for *OXR1* and *TLDC1*. Moreover, *OXR1*, *TLDC1*, and *TBC1D24* mRNA levels were found to positively correlate with the severity of ASD. This suggests that the overexpression of these genes is an attempt to cope with the OS associated with ASD, which in turn is greater in subjects with more severe cognitive impairment [24]. However, the presence of oxidative stress markers in our ASD patients [24] indicates that the upregulation of these TLDc domain-containing genes does not suffice to counteract OS in ASD.

The mechanisms responsible for the upregulation of *OXR1* and *TLDC1* in ASD remains to be elucidated. MicroRNAs (miRNAs) are important post-transcriptional regulators that affect both the stability and the translation of mRNAs. Among the numerous miRNAs identified as modulators of TLDc domain-containing mRNAs, we focused on those that bear some relation to the OS response and ASD. In diabetic retinopathy, miR-200b targets *OXR1*, inducing a decrease in both OXR1 mRNA and protein and exacerbating oxidative damage [36]. 

Moreover, notwithstanding that miR-200b is located in autism-associated CNV loci [58], its expression in ASD has been so far completely unexplored. Our results show no difference in the miR-200b-3p levels in ASD subjects compared to NTD subjects, suggesting that it does not affect *OXR1* upregulation in ASD PMBCs. miR-32-5p is another modulator of TLDc domain-containing mRNAs [46]; its expression had been found to be downregulated in the saliva of ASD children [59,60]. However, our data did not indicate a difference in miR-32-5p expression levels between ASD and NTD children, thus excluding its regulatory role in our model. These results can be explained in light of the known tissue-specific expression of miRNAs [61]. Beside miRNAs, lncRNAs are a further class of gene expression modulators. The lncRNA TUG1 was suggested as a positive regulator of *TLDC1* in knockdown cell models [50]. Consistent with the upregulation of TUG1 in the peripheral blood of ASD patients previously observed by Sayad et al. [49], we found that its level was increased in ASD children compared to NTD children. This result reinforces the implication of lncRNA TUG1 in autism and can be related to the increase in the *TLDC1* expression level observed in ASD.

Although TLDc family proteins play a role in the oxidative response, their exact mechanism of action has not been elucidated. Our data show the upregulation of two TLDc-domain containing RNAs in ASD subjects along with the concomitant increase in NFR2 mRNA levels [51]. Most of the studies currently available concern OXR1, reporting that its depletion prevents the expression of NRF2-dependent antioxidant genes such as glutathione peroxidase 2 (GPX2) and heme oxygenase 1 (HO-1/HMOX1) [62]. In this context, the proposed mechanism of OXR1 action is its interaction with Keap-1 (kelch like ECH associated protein 1), which controls NRF2 degradation [63]. On the one hand, it is well known that an increase in NRF2 transcription does not necessarily result in an increase in NRF2-mediated upregulation of antioxidant proteins since it requires NRF2 escape from degradation and its protein translocation to the nucleus, which depends on several factors [64]. On the other hand, the transcriptional increase in OXR1 may not suffice to inhibit Keap-1 activity. Therefore, the upregulation of *NRF2* and *OXR1* here described may not be efficient enough to mount an effective antioxidant response, as shown by the presence of oxidative damage in ASD [24,65,66]. However, one should not overlook the fact that the transcriptional upregulation of TLDc domain-containing RNAs and their correlation with NRF2 transcriptional upregulation is one of the first pieces of direct evidence of the presence of an antioxidant response in ASD.

Oxidative stress may derive from different sources, including the activation of inflammatory pathways, at the level of the central nervous system by the involvement of microglia [67,68,69], at the intestinal level through alterations in the microbiota composition and the intestinal barrier, or in other body districts [70]. These alterations have been described in ASD patients [15]. TLDc domain-containing proteins may control neuroinflammation and the immune response [62]. In our model, the positive correlation between the expression of some relevant inflammation genes and that of TLDc family genes suggests a protective role of these proteins against the OS generated by inflammation. The correlation panel regarding TLDc domain-containing genes and inflammation/oxidation-related gene expression shows that each TLDc gene has a different correlation pattern. For instance, the expression of *COX2* correlates with the expression of *OXR1* and *NCOA7* but not with the expression of *TLDC1* and *TBC1D24*. It is conceivable that this depends on the additional motifs which confer to each member additional properties and influence the functional effects of the TLDc domain [31]. Moreover, the presence of different inflammation and oxidative stimuli could specifically activate the expression of different TLDc genes [71]. Because of the relatively small sample size of the present study, these assumptions need to be validated in a larger sample of patients and do not allow further speculation. 

## 4. Materials and Methods

### 4.1. Ethics Statement

The present study was carried out in accordance with the Declaration of Helsinki guidelines, and the protocol was approved by the Local Ethical Committee (Azienda USL Bologna, CE 10020- n.30, 06/04/2010 prot 45424/10-03). Signed informed consent was obtained from all parents and, whenever possible, from children through forms showing pictures aimed at clarifying the clinical study prior to participation.

### 4.2. Sample Collection and mRNA Extraction

The experiments performed in this study were carried out with total RNA samples obtained from PBMCs of 16 children diagnosed with nonsyndromic ASD [12 males and 4 females, aged (mean ± standard deviation-SD) 6.2 ± 1.8 years] and 16 NTD children [11 males and 5 females, aged (mean ± SD) 7.6 ± 2.1 years] recruited by the Child Neuropsychiatry Unit of the Bellaria Hospital (IRCCS Istituto delle Scienze Neurologiche di Bologna). Demographic parameters and clinical features of the ASD patients are shown in Table S1 and are a subset of those published in Ghezzo et al. [24]. Briefly, children underwent an accurate clinical evaluation, including electroencephalography (recorded both awake and sleeping), cerebral magnetic resonance imaging, CGH array, and molecular assay for Fragile X and MECP2. No anomaly in these parameters was observed. None of the ASD subjects had active epilepsy at the time of blood sampling. As reported in Ghezzo et al. [24], ASD children were diagnosed using the Diagnostic and Statistical Manual of Mental Disorders, Fourth Edition, Text Revision (DSM-IV-TR) criteria [72], Autism Diagnostic Observation Schedule (ADOS), and Childhood Autism Rating Scale (CARS). Notably, according to the Diagnostic and Statistical Manual of Mental Disorders, Fifth Edition (DSM-5) criteria [1], all children were affected by ASD. Control children, recruited in the same local community, were assessed to exclude cognitive, learning, and neuropsychiatric problems. Subjects did not consume any dietary supplement in the four months before the biochemical and clinical evaluations and were free of any inflammatory problems or infections. 

The present study was part of a larger project, where the expression of several genes and other parameters relative to oxidative stress, membrane lipids, enzymatic activities, and inflammation markers were evaluated. Blood (9 mL) was collected in Na_2_-EDTA vacutainers, and PBMCs were separated by layering blood over a Ficoll–Paque gradient (Sigma-Aldrich, St. Louis, MO, USA). To extract total RNA, PBMCs were lysed in 1 mL Trizol^®^ Reagent (Invitrogen, Waltham, MA, USA) according to the manufacturer’s instructions [73]. RNA was quality controlled as previously reported [20,74].

### 4.3. cDNA Synthesis and Quantitative Real-Time PCR (qPCR)

cDNA synthesis for mRNA and lncRNA expression analysis was performed by reverse transcribing 800 ng of total RNA using the iScript™ cDNA Synthesis Kit (Bio-Rad Laboratories, Hercules, CA, USA). The expression of both mRNAs and lncRNAs was analyzed by qPCR using SsoAdvanced™ Universal SYBR^®^ Green Supermix (Bio-Rad Laboratories, Hercules, CA, USA) according to the datasheet instructions and employing the CFX96 real-time thermal cycler (Bio-Rad Laboratories, Hercules, CA, USA). Primer sequences (Table 3) were custom designed using the Primer Blast and AMPLIFY free software and, whenever possible, they spanned an exon–exon junction. For each gene, the primers were designed in order to recognize all the isoforms described in the Gene NCBI database “https://www.ncbi.nlm.nih.gov/gene/” (accessed on 23 September 2023). Primers were purchased from Sigma Genosys (Sigma, St. Louis, MO, USA). Actin-beta (*ACTB*) and glyceraldehyde-3-phosphate dehydrogenase (*GAPDH*) were used as reference genes. The stability of housekeeping genes was validated according to the method described by Hellemans et al. [75].

Data were analyzed with the 2^−ΔΔCt^ method [76] using the CFX Manager (Bio-Rad Laboratories, Hercules, CA, USA) and the qBase plus software (Biogazelle). Data are expressed as the fold change ± the confidence interval. 

To evaluate miRNA expression, 5 ng/µL of total RNA was reverse transcribed using the miRCURY LNA RT Kit (Qiagen, Valencia, CA, USA) according to the manufacturer’s instructions. Quantitative PCR assays were carried out using the CFX96 real-time thermal cycler (Biorad, Bio-Rad Laboratories, Hercules, CA, USA) and miRCURY LNA SYBR Green Master Mix (Qiagen, Valencia, CA, USA). The reactions were set up in a 10 μL reaction volume containing 3 μL cDNA diluted 60-fold in RNase free water and miRCURY LNA miRNA PCR assay primers, which were purchased from Qiagen (Valencia, CA, USA). The miRNA primer assay IDs are shown in Table 4. miR-16-5p and U6 snRNA were used as reference genes for normalization purposes [77,78]. Data were analyzed as described above.

### 4.4. Statistics

Normality tests were applied to all gene and miRNA expression data as well as to clinical features. All genes and miRNAs passed the normality test (D’Agostino and Pearson test); therefore, an appropriate parametric test (*unpaired t-test*) was used to compare ASD and NTD data. The correlation between TLDc gene expression and clinical features was determined using the parametric Pearson correlation coefficient (r). The Pearson correlation test was also used to correlate the expression of TLDc genes and inflammation/oxidation-related genes. Correlations were considered significant at *p* ≤ 0.05. To account for multiple tests, we used FDR. FDR-corrected *p*-values (pFDR) were evaluated separately for (a) comparisons of TLDc gene expression, miRNAs, and TUG1 lncRNA in ASD and NTD subjects; (b) correlations between the expression of TLDc genes and CARS global scores in ASD children; and (c) correlations between the expression of TLDc genes and inflammation/oxidation-related genes in ASD children. 

## 5. Conclusions

To our knowledge, this is the first pilot study that describes the expression profile of genes coding for TLDc domain-containing proteins in PBMCs from ASD children. OXR1 and TLDC1 were upregulated in ASD patients, and their expression levels correlated with the severity of symptoms of the condition and with the expression levels of inflammation/oxidative stress genes. These findings may provide new insights into the involvement of TLDc family genes in the modulation of OS in ASD, suggesting a novel biological pathway in OS response and paving the way to discover new potential biomarkers for diagnosis and therapy. A limitation of this study is that we have not quantified the different gene isoforms of TLDc genes, which may depend, among other factors, on the presence of specific inflammatory or oxidant stimuli. Another limitation pertains to the fact that we did not have enough biological material to allow the evaluation of TLDc domain-containing proteins in PBMCs; thus, we were unable to confirm whether the mRNA upregulation resulted in an increase in protein expression. These aims will be pursued in future studies. Moreover, a larger sample of patients stratified by oxidative and inflammatory features, as well as by microbiota alterations, will be required to validate our findings and to better understand the molecular mechanisms controlling the expression and function of the TLDc domain-containing proteins. Consequently, we are planning to extend the study to ASD cellular and animal models, in which the expression of TLDc domain-containing proteins can be modulated.

## Figures and Tables

**Table 1:** Correlation between gene expression of TLDc domain-containing proteins and Childhood Autism Rating Scale (CARS) global scores in autism spectrum disorder (ASD) children.

TLDc Genes	Correlations of Gene Expression with CARS Global Score
*OXR1*	r = 0.6055 *p* = 0.0129 pFDR = 0.0258
*TLDC1*	r = 0.5421 *p* = 0.0301 pFDR = 0.0401
*NCOA7*	NS
*TBC1D24*	r = 0.7613 *p* = 0.0006 pFDR = 0.0024

CARS, Childhood Autism Rating Scale; *OXR1*, oxidation resistance 1; *TLDC1*, TBC/LysM-associated domain containing 1; *NCOA7*, nuclear receptor coactivator 7; *TBC1D24*, TBC1 domain family member 24; r, Pearson coefficient correlation; pFDR, Benjamini and Hochberg false discovery rate corrected *p*-values; NS, not significant.

**Table 2:** Correlations between expression levels of TLDc domain-containing genes and inflammation/oxidation-related genes.

TLDc Genes	Inflammation/Oxidation-Related Genes					
	*IL1B*	*TNF-Alpha*	*COX2*	*AHR*	*NRF2*	*PRDX2*
*OXR1*	r = 0.5082	NS	r = 0.5727	NS	NS	NS
	*p* = 0.0444		*p* = 0.0204			
	pFDR = 0.0999		pFDR = 0.0999			
*TLDC1*	r = 0.4926 *p* = 0.0525 pFDR = 0.0787	r = 0.5401 *p* = 0.0308 pFDR = 0.0616	NS	r = 0.5885 *p* = 0.0165 pFDR = 0.0495	r = 0.6189 *p* = 0.0106 pFDR = 0.0495	NS
*NCOA7*	NS	NS	r = 0.5302	NS	r = 0.525	NS
			*p* = 0.0346		*p* = 0.0368	
			pFDR = 0.1104		pFDR = 0.1104	
*TBC1D24*	r = 0.6682 *p* = 0.0047 pFDR = 0.0094	r = 0.6249 *p* = 0.0096 pFDR = 0.0144	NS	r = 0.8831 *p* =< 0.0001 pFDR = 0.0006	r = 0.6972 *p* = 0.0027 pFDR = 0.0081	NS

*IL1B*, interleukin 1 beta; *TNF-alpha*, tumor necrosis factor-alpha; *COX2*, cyclooxygenase 2; *AHR*, aryl hydrocarbon receptor; *PRDX2*, peroxiredoxin 2; *NRF2*, nuclear factor erythroid 2-related factor 2; r, Pearson coefficient correlation; pFDR, Benjamini and Hochberg false discovery rate corrected *p*-values; NS, not significant.

**Table 3:** Primer sequences and amplicon length of the genes analyzed by quantitative real-time PCR (qPCR).

Gene ID	GENE	Left Primer	Right Primer	Amplicon Length (bp)
55074	*OXR1*	acaggtttttggtgcgttagc	ccaaagcgcaaattctcctcc	193
57707	*TLDC1*	tgtacacacacacgggctac	acatccacccaaagcccaaa	118
135112	*NCOA7*	gctctaccggaaatcggcat	gaaaagtttcgcctgtgccat	137
57465	*TBC1D24*	ttgctcatcaagaccacgca	cttgatcaccacccactcgt	165
140711	*C20Orf118*	aagggagacttggattcactgatg	tgtttacaaactccctctcctgc	246
55000	*TUG1*	gcaccagattccagaaaaggc	aagggcttcatggccaca	104
60	*ACTB* ^1^	tgtggcatccacgaaactac	tgatcttgatcttcattgtgct	175
2597	*GAPDH* ^1^	ggcctccaaggagtaagacc	ctgtgaggaggggagattca	130

*OXR1*, oxidation resistance 1; *TLDC1*, TBC/LysM-associated domain containing 1; *NCOA7*, nuclear receptor coactivator 7; *TBC1D24*, TBC1 domain family member 24; *C20Orf118,* TBC/LysM-associated domain containing 2; *TUG1*, taurine up-regulated 1; *ACTB*, actin beta; *GAPDH*, glyceraldehyde-3-phosphate dehydrogenase. ^1^ *ACTB* and *GADPH* were used as reference genes for normalization purposes.

**Table 4:** Correspondence of gene name of noncoding RNAs (ncRNA) evaluated and the Qiagen GeneGlobe ID of the miRCURY LNA miRNA PCR assay.

ncRNA	Qiagen GeneGlobe ID
miR-200b-3p	YP00206071
miR-32-5p	YP00204792
miR-16-5p ^1^	YP00205702
U6 snRNA ^1^	YP02119464

^1^ miR-16-5p and U6 snRNA were used as reference genes for normalization purposes.

## Data Availability

Data used in this study are available from the corresponding author on reasonable request.

## Supplementary Materials

The following supporting information can be downloaded at: https://www.mdpi.com/article/10.3390/ijms242115802/s1.
