# Supplementary material for: TLDc Domain-Containing Genes in Autism Spectrum Disorder: New Players in the Oxidative Stress Response
Source: Int J Mol Sci. 2023 Oct 31;24(21):15802. doi: 10.3390/ijms242115802 (PMC10647648; doi:10.3390/ijms242115802)
Supplement: Supplementary file 1 [file ijms-24-15802-s001.zip › Table S1.pdf]

Table S1: Demographic and clinical features of the Autism Spectrum Disorder children group

No.	Gender	Age (months)	Age of onset (months)	Cognitive/ developmental impairment	DSM-IV-TR diagnosis	ADOS global score	ADOS diagnosis	CARS global score
1	M	66	≤12	Moderate	PDD-Au	18	Au	40.5
2	M	74	≤12	Severe	PDD-Au	21	Au	42
3	M	64	≤12	Moderate	PDD-Au	16	Au	35
4	M	103	13-18	Moderate	PDD-Au	19	Au	44.5
5	M	61	≤12	Severe	PDD-Au	21	Au	46
6	M	71	13-18	Severe	PDD-Au	22	Au	41
7	M	142	≤12	Severe	PDD-Au	22	Au	44.5
8	M	66	13-18	Moderate	PDD-Au	22	Au	40.5
9	F	66	13-18	Borderline IQ	PDD-NOS	15	Au	41.5
10	M	74	13-18	Severe	PDD-Au	22	Au	42.5
11	F	66	13-18	Severe	PDD-Au	22	Au	43.5
12	M	66	13-18	Mild	PDD-NOS	14	Au	34
13	M	102	13-18	Mild	PDD-Au	22	Au	36.5
14	F	110	25-30	Moderate	PDD-Au	15	Au	47.5
15	M	80	≤12	Normal IQ	PDD-Au	19	Au	36.5
16	F	65	≤12	Mild	PDD-NOS	17	Au	31.5

PDD: Pervasive Developmental Disorder; ; Au: Autism; PDD-NOS: Pervasive Developmental Disorder -Not Otherwise Specified.
